# High sensitivity and specificity of 4D-CTA in the detection of cranial arteriovenous shunts

**DOI:** 10.1007/s00330-019-06234-4

**Published:** 2019-05-14

**Authors:** Matthijs in ’t Veld, Rolf Fronczek, Marlise P. dos Santos, Marianne A. A. van Walderveen, Frederick J. A. Meijer, Peter W. A. Willems

**Affiliations:** 1 Department of Radiology, Leiden University Medical Center, Leiden, The Netherlands; 2 Department of Neurology, Leiden University Medical Center, Leiden, The Netherlands; 3 Sleep-Wake Centre, SEIN, Heemstede, The Netherlands; 4 Department of Medical Imaging, University of Ottawa & Ottawa Hospital Research Institute, The Ottawa Hospital, Ottawa, Canada; 5 Department of Radiology, Radboud University Medical Center Nijmegen, Nijmegen, The Netherlands; 6 Department of Neurosurgery, Utrecht University Medical Center, Internal Postage G03.124, PO-box 85500, 3584 CX Utrecht, The Netherlands

**Keywords:** Four-dimensional computed tomography, Angiography, Brain imaging, Dural arteriovenous fistula, Arteriovenous malformations

## Abstract

**Purpose:**

In a prospective cohort study, we evaluated the diagnostic accuracy of time-resolved CT angiography (4D-CTA) compared to digital subtraction angiography (DSA) for detecting cranial arteriovenous shunts.

**Material and methods:**

Patients were enrolled if a DSA had been ordered querying either a dural arteriovenous fistula (dAVF) or a cerebral arteriovenous malformation (bAVM). After enrolment, both a DSA and a 4D-CTA were performed. Both studies were evaluated using a standardized form. If a dAVF or bAVM was found, its classification, angioarchitectural details, and treatment options were recorded.

**Results:**

Ninety-eight patients were enrolled and 76 full datasets were acquired. DSA demonstrated a shunting lesion in 28 out of 76 cases (prevalence 37%). 4D-CTA demonstrated all but two of these lesions (sensitivity of 93%) and produced one false positive (specificity of 98%). These numbers yielded a positive predictive value (PPV) of 96% and a negative predictive value (NPV) of 96%. Significant doubt regarding the 4D-CTA diagnosis was reported in 6.6% of all cases and both false-negative 4D-CTA results were characterized by such doubt.

**Conclusions:**

4D-CTA has very high sensitivity and specificity for the detection of intracranial arteriovenous shunts. Based on these results, 4D-CTA may replace DSA imaging as a first modality in the diagnostic workup in a large number of patients suspected of a cranial dAVF or bAVM, especially if there is no doubt regarding the 4D-CTA diagnosis.

**Key Points:**

*• 4D-CTA was shown to have a high diagnostic accuracy and is an appropriate, less invasive replacement for DSA as a diagnostic tool for cranial arteriovenous shunts in the majority of suspected cases.*

*• Doubt regarding the 4D-CTA result should prompt additional DSA imaging, as it is associated with false negatives.*

*• False-positive 4D-CTA results are rare, but do exist.*

## Introduction

Intracranial arteriovenous malformations (AVMs) represent abnormal connections between arteries and veins. They may be divided into brain (pial) AVMs (bAVMs) and dural AVMs depending on whether the feeders are pial or dural arteries. bAVMs may be further divided into nidus-type AVMs, if there is a tangle of abnormal vessels, and arteriovenous fistulas (AVFs), if there is no intervening network [1, 2]. Since dural AVMs do not have a true nidus, they may also be called dural AVFs (dAVFs). dAVFs may drain solely through venous sinuses in which case they do not represent a risk of hemorrhage. They may also drain through or reflux into pial veins in which case they do represent a risk of hemorrhage [3, 4]. bAVMs and dAVFs may be asymptomatic or present with a wide variety of symptoms such as focal neurological deficits, headache, bruit, or seizures. Angiographically, all of these lesions are characterized by early venous filling caused by the lack of passage of blood through a normal capillary network.

Although computed tomographic angiography (CTA) and magnetic resonance angiography (MRA) can demonstrate many characteristics of an arteriovenous shunt, digital subtraction angiography (DSA) is the gold standard to prove the existence of the shunt and distinguish it from mimicking lesions [5–7]. Furthermore, DSA can identify the feeding arteries and venous draining patterns [8]. Unfortunately, DSA is time-consuming, expensive, and not without risks, due to its invasive nature. Although DSA may be considered to be a safe diagnostic procedure in experienced hands, it carries a risk of (silent) embolic events, especially in patients with vascular risk factors [9]. Serious complications are rare, but can result in transient or permanent neurological damage [10–12].

Whole-head time-resolved CTA (4D-CTA) allows visualization of blood flow dynamics in cranial vessels with the first pass of an intravenous contrast bolus. 4D-CTA is less expensive and less time-consuming than conventional DSA [13, 14]. Furthermore, it is non-invasive and carries no risk of thromboembolic complications. Earlier small studies of selected cases suggested a role for 4D-CTA in the diagnostic workup, treatment planning, and follow-up of cranial dAVFs and bAVMs [15–18]. The current study aims to determine the true diagnostic value of 4D-CTA for such lesions in a non-selected patient population.

## Material and methods

### Patient data and data collection

The study protocol was approved by our institutional research ethics board in April 2010 and was conducted according to the principles of the Declaration of Helsinki.

At the two participating institutions (Leiden University Medical Centre and Radboud University Medical Centre), all patients were prospectively recruited from October 2010 until January 2016 in a tertiary care setting. Patients were included if they were 18 years or older, the presence of a dAVF or bAVM was suspected, and a DSA was requested to confirm this diagnosis. Exclusion criteria were a previous diagnosis of dAVF or bAVM with time-resolved imaging, current treatment for diabetes mellitus, renal failure (baseline eGFR < 50 ml/min), or a known allergy to iodinated contrast agents. After obtaining informed consent, patients underwent both DSA and 4D-CTA imaging.

### DSA examination

All DSA examinations were performed using standard biplane fluoroscopy (Infinix®, Toshiba Medical Systems; or Allura Xper®, Philips Healthcare). A routine angiographic protocol was performed and generally consisted of bilateral injections of the internal and external carotid arteries and at least one vertebral artery. During each injection, anterior-posterior (AP) and lateral projections were obtained at 6 frames/s.

### 4D-CTA examination

4D-CTA examination was performed using an Aquilion ONE multidetector CT scanner (Toshiba Medical Systems), equipped with 320 × 0.5 mm detector rows covering 16 cm of volume per rotation. Imaging was performed in a manner previously described [14–16, 19].

Although acquisition protocols could vary, the study protocol required a minimum rotation speed of 1 Hz and a maximum reconstruction interval of 1 s during the continuous phase, i.e., the phase of continuous acquisition during which the contrast bolus traveled from arterial to venous vasculature. The highest rotation speed and shortest reconstruction interval used were 2 Hz and 0.3 s, respectively [20].

Typically, the entire study would be as follows. An intravenous infusion of 60 ml non-ionic contrast was followed by 29 ml of saline. Subsequently, the following volumes were acquired: a mask volume, two volumes before contrast medium could reach the cranial vessels, the continuous phase as detailed above, and three volumes during venous “wash-out.” The mask volume was subtracted from all subsequent volumes which were then stored in DICOM files.

Depending on the reconstruction interval during the continuous phase, a total of 22 to 51 volumes were reconstructed. These volumes were used to reconstruct time-resolved (arterial to venous) maximum intensity projection series in AP, lateral, and several oblique viewing angles. Typically, the oblique reconstructions were projected at 30°, 45°, and 60°. Two series were added in the arterial phase (without temporal resolution) in which the volume rotated around an axial axis (“spin”) and a lateral axis (“tumble”), again using maximum intensity projections. Finally, a slice stack was generated using time-averaged intensities. If necessary, additional post-processing options were available, but were generally not requested.

### Angiographic evaluation

One reader at each participating institution read the DSA and 4D-CTA images, locally. This was an experienced neuroradiologist (FJAM) at one institution and an experienced neurointerventionalist (PWAW) at the other. A standard score sheet was used to score all relevant aspects of the imaging. To blind the readers for the DSA result at the time of 4D-CTA evaluation, the 4D-CTA was always reviewed before the DSA. The protocol allowed this to be done after both studies had already been acquired, as long as the reader was not aware of the DSA result.

The scoring sheet consisted of the following items: the presence of a vascular lesion (“dAVF,” “bAVM,” or “other”); the certainty of the diagnosis using a 3-point scale (“absolutely certain,” “sufficiently certain,” or “significant doubt”); the overall quality of the images in case of 4D-CTA, using a 3-point scale (“good,” “moderate,” “poor”); the classification according to either Borden for dAVFs or Spetzler-Martin for bAVMs; the type of fistulous area in case of a dAVF (“focal” or “diffuse”) and the types of feeders that could be identified. The latter assessment consisted of “posterior circulation” and/or “anterior circulation” for bAVMs and “external carotid” and/or “internal carotid” and/or “vertebrobasilar” for dAVFs. Finally, the reader was asked to record whether the 4D-CTA study would have been sufficient to determine the best treatment strategy and whether DSA would have been required, additionally, prior to such treatment.

With regard to the first item on the scoring sheet, i.e., the diagnosis, pial AVFs were classified as bAVMs, since the distinction between a very small nidus (micro-AVM) and a pial AVF may be difficult on imaging and their hemodynamic and clinical characteristics are very similar.

### Interobserver analysis

Following the initial analysis, a representative subset of 30 4D-CTA records was selected. These were independently evaluated by a third experienced neurointerventional radiologist (MAAVW) in exactly the same way as the initial analysis. This radiologist was blinded for both the prior 4D-CTA assessments and the DSA results.

### Complications of DSA

For all cases with complete datasets, the hospital records were screened for complications relating to the DSA.

### Statistical analysis

Sensitivity, specificity, and positive and negative predictive values for 4D-CTA were calculated using SPSS® software (version 23.0, IBM Corp.), with DSA as the golden standard. For the interobserver analysis, kappa values were calculated for the diagnosis (“no diagnosis,” “dAVF,” or “bAVM”), Borden classification, and Spetzler-Martin classification.

## Results

### Inclusion

**Fig. 1:**
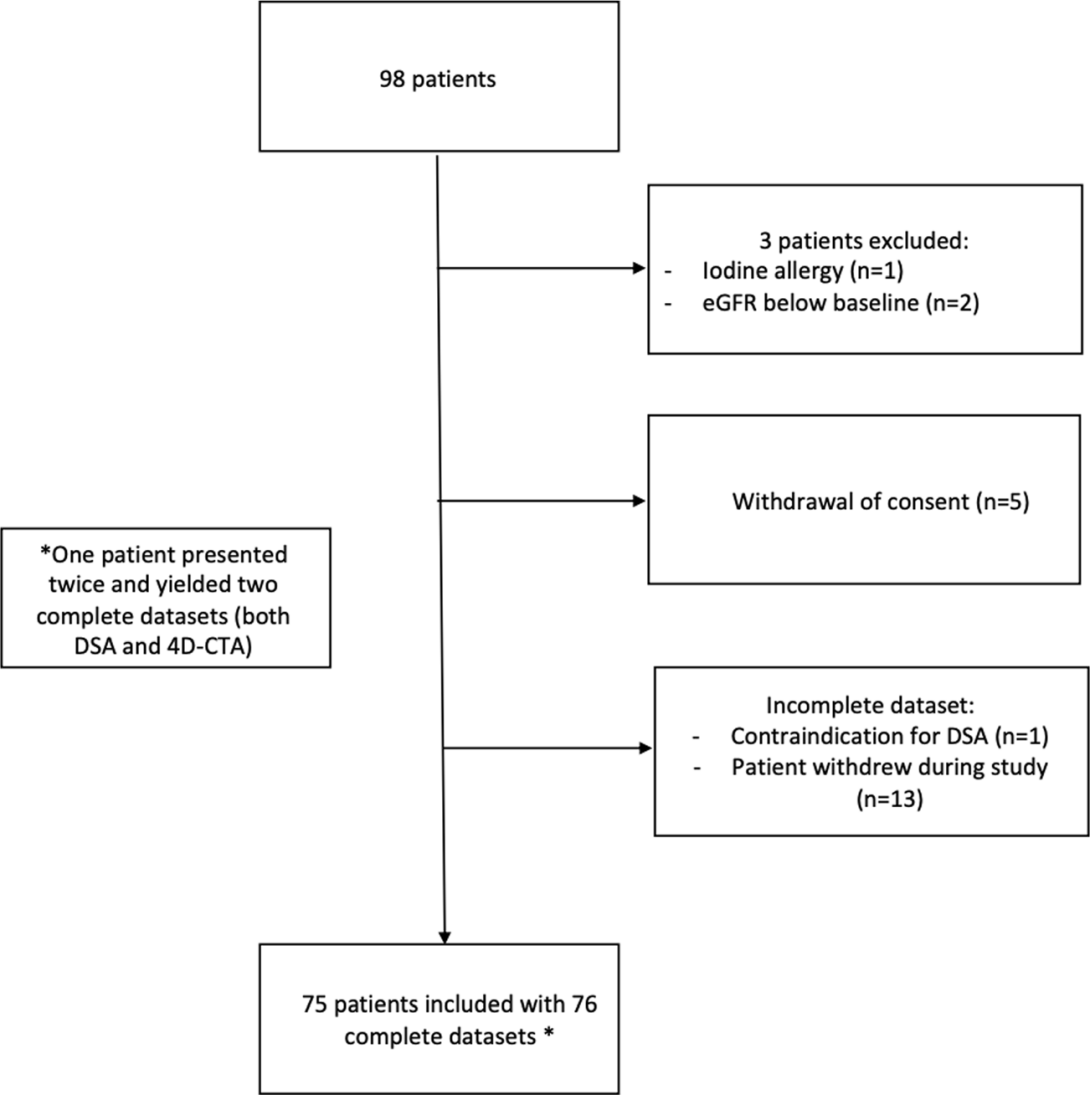
Flowchart of patient inclusion

Between October 2010 and January 2016, 98 patients with a clinical suspicion of a cranial arteriovenous shunt were included. Seventy-six complete datasets, containing both a DSA and a 4D-CTA, were obtained in 75 patients. The average interval (± SD) between both studies was 32.6 days (± 43.7). In 71.1% of the cases, the second study was acquired within 4 weeks of the first. One patient was enrolled twice on two unrelated occasions, first due to hemorrhage and 3 months later due to tinnitus. This yielded two complete datasets which were analyzed independently (see Fig. 1 for the patient inclusion details). Table 1 shows the baseline characteristics and presenting symptoms of these patients.

**Table 1:** Patient characteristics

Characteristic		Number	Percentage
Age (mean ± SD)		53.1 ± 12.6	
Sex	Male	35	46.1
	Female	41	53.9
Presenting symptom	Hemorrhage	12	15.6
	Focal neurologic deficits	2	2.6
	Tinnitus	41	53.2
	Bruit	6	7.8
	Seizure	3	3.9
	Headache	4	5.2
	Other symptoms	8	11.7

*SD*, standard deviation

### Prior imaging

Prior imaging was performed in 61 cases (80.3%), consisting of CT (17), contrast-enhanced CT (6), CTA (13), MR (20), contrast-enhanced MR (20), and/or MRA (28). In 35 cases (57.4%), this prior imaging was reported to have contributed to the suspicion of an arteriovenous shunt (dAVF or bAVM). Since we aimed to determine the value of 4D-CTA in a realistic setting, this prior imaging was available to the person reading the DSA or 4D-CTA.

### Diagnostic value of 4D-CTA

The imaging results are listed in Table 2. DSA demonstrated an arteriovenous shunt in 28 out of 76 datasets (prevalence of 36.8%). This was a bAVM in 9 and a dAVF in 19 cases. 4D-CTA demonstrated all but two of these shunting lesions. Of the 9 bAVM patients, 8 were correctly diagnosed (88.9%) and one was reported normal by 4D-CTA (false negative). Of the 19 dAVF patients, 17 were correctly diagnosed (89.5%), one was reported as a bAVM (misclassified), and one was reported normal by 4D-CTA (false negative). Of the 48 patients where DSA did not demonstrate a shunt, 47 were correctly negative (97.9%) and one was incorrectly diagnosed with a Borden type 1 dAVF with 4D-CTA (false positive). The resulting 4D-CTA test characteristics are shown in Table 3.

**Table 2:** Diagnosis found by 4D-CTA

Diagnosis by 4DCTA	No.	Diagnosis by DSA	No.
AVM	9	Confirmed AVM	8
		dAVF	1
dAVF	18	Confirmed dAVF	17
		No diagnosis	1
Other diagnosis	8	Confirmed “Other diagnosis”	8
No diagnosis	41	Confirmed “No diagnosis”	37
		AVM	1
		dAVF	1
		Other diagnosis	2
	*n* = 76		*n* = 76

*4D-CTA*, time-resolved computer tomography angiography; *DSA*, digital subtraction angiography; *dAVF*, dural arteriovenous fistula; *AVM*, arteriovenous malformation

**Table 3:** Sensitivity, specificity, positive predictive value, and negative predictive value of 4D-CTA in diagnosing cerebral shunting lesions

		DSA		
		Shunt present	Shunt absent	
4DCTA	Test positive	26	1	27
	Test negative	2	47	49
		28	48	76

Sensitivity 92.9%, positive predictive value 96.3%

Specificity 97.9%, negative predictive value 95.9%

*4D-CTA*, time-resolved computer tomography angiography; *DSA*, digital subtraction angiography

Results of 4D-CTA and DSA regarding further details of the arteriovenous shunts are provided in Table 4. Although 4D-CTA agreed with DSA on Borden and Spetzler-Martin classification, there was less agreement regarding further details such as feeding arteries and delineation of the fistulous area.

**Table 4:** Characteristics of the shunting lesions confirmed by 4D-CTA

Diagnosis	Characteristics	DSA (golden standard)	Correctly identified by 4D-CTA (%)
dAVF	Number of dAVFs	19	17^a^
	Borden I	10	10(100%)
	II	2	2 (100%)
	III	7	5 (71%)
	Feeding branches		
	ECA	10	7 (70%)
	ICA	1	1 (100%)
	VA	1	1 (100%)
	Multiple feeding territories	7	0 (0%)
	Fistulous area		
	Focal area	12	6 (50%)
	Diffuse area	7	3 (43%)
AVM	Number of AVMs	9	8^b^
	SM size < 3 cm	9	8 (89%)
	Eloquent Non-eloquent	5 4	4 (80%) 4 (100%)
	Superficial drainage Deep drainage	4 5	3 (75%) 5 (100%)
	Feeding branches		
	Anterior circulation	5	4 (80%)
	Posterior circulation	2	1 (50%)
	Both	1	0 (0%)

^a^4D-CTA missed a dAVF in one patient and one dAVF was misclassified

^b^4D-CTA missed an AVM in one patient

*dAVF* dural arteriovenous fistula, *AVM* arteriovenous malformation, *DSA* digital subtraction angiography, *4D-CTA* time-resolved computer tomography angiography, *ECA* external carotid artery, *ICA* internal carotid artery, *VA* vertebral artery, *SM* Spetzler-Martin

With regard to the locations of the 19 dAVFs, 13 were located at the level of the transverse or sigmoid sinus, one at the level of the torcular herophili, one at the free margin of the tentorium cerebelli, one in the parietal region, draining directly into a cortical vein, one on the cerebellar convexity. The final two were cavernous sinus dAVFs.

In 10 cases, DSA showed an abnormality other than a shunt: two dural sinus stenoses, three developmental venous anomalies, two arterial aneurysms, one venous aneurysm, one calcifying tumor, and one internal carotid artery dissection. Eight of these (80%) were also correctly reported on 4D-CTA and two were not reported: one developmental venous anomaly and one venous aneurysm.

### Certainty and image quality in 4D-CTA

The image quality was reported to be “good” in 55 (73.7%), “moderate” in 15 (19.7%), and “poor” in 5 cases (6.6%). Comparing DSA with 4D-CTA, the level of certainty regarding the diagnosis was as follows: 72 versus 22 for “absolutely certain,” 4 versus 49 for “sufficiently certain,” and 0 versus 5 for “significant doubt.”

For the two cases with a false-negative 4D-CTA result, the image quality was reported to be “good” in one and “moderate” in the other and the level of certainty regarding the 4D-CTA diagnosis was “significant doubt” in both. In other words, out of five 4D-CTAs with significant doubt, two diagnoses were indeed false negative (40%). Thus, if we removed all cases with significant doubt about the diagnosis (6.6% of the 4D-CTAs), the diagnostic value of the 4D-CTA would increase, with a sensitivity of 100%. The reasons for reduced image quality were not addressed in the score sheets but, generally, consist of motion artifacts and or poor contrast bolus timing.

### Treatment strategy

In 16 of the 27 cases (59.3%) where 4D-CTA demonstrated a bAVM or dAVF, the 4D-CTA study was thought to be sufficient to determine the best applicable treatment strategy. In the other 11 cases, additional DSA was still deemed necessary for the treatment planning.

### Complications of DSA

Ten patients (13.2%) reported temporary subjective symptoms such as slightly blurred vision (6), headaches (3), or a combination of the two (1).

Two patients (2.6%) experienced transient neurological signs: one mild ataxia and one hemiparesis. Both recovered quickly and completely.

Eight cases (10.5%) suffered from an inguinal hematoma. In four of these patients, Angio Seal (St. Jude Medical) had been used as closure system.

No permanent deficits, systemic complications, or mortality were recorded and none of the patients required readmission after discharge.

### Details of the four cases that were incorrectly classified by 4D-CTA

**Fig. 2:**
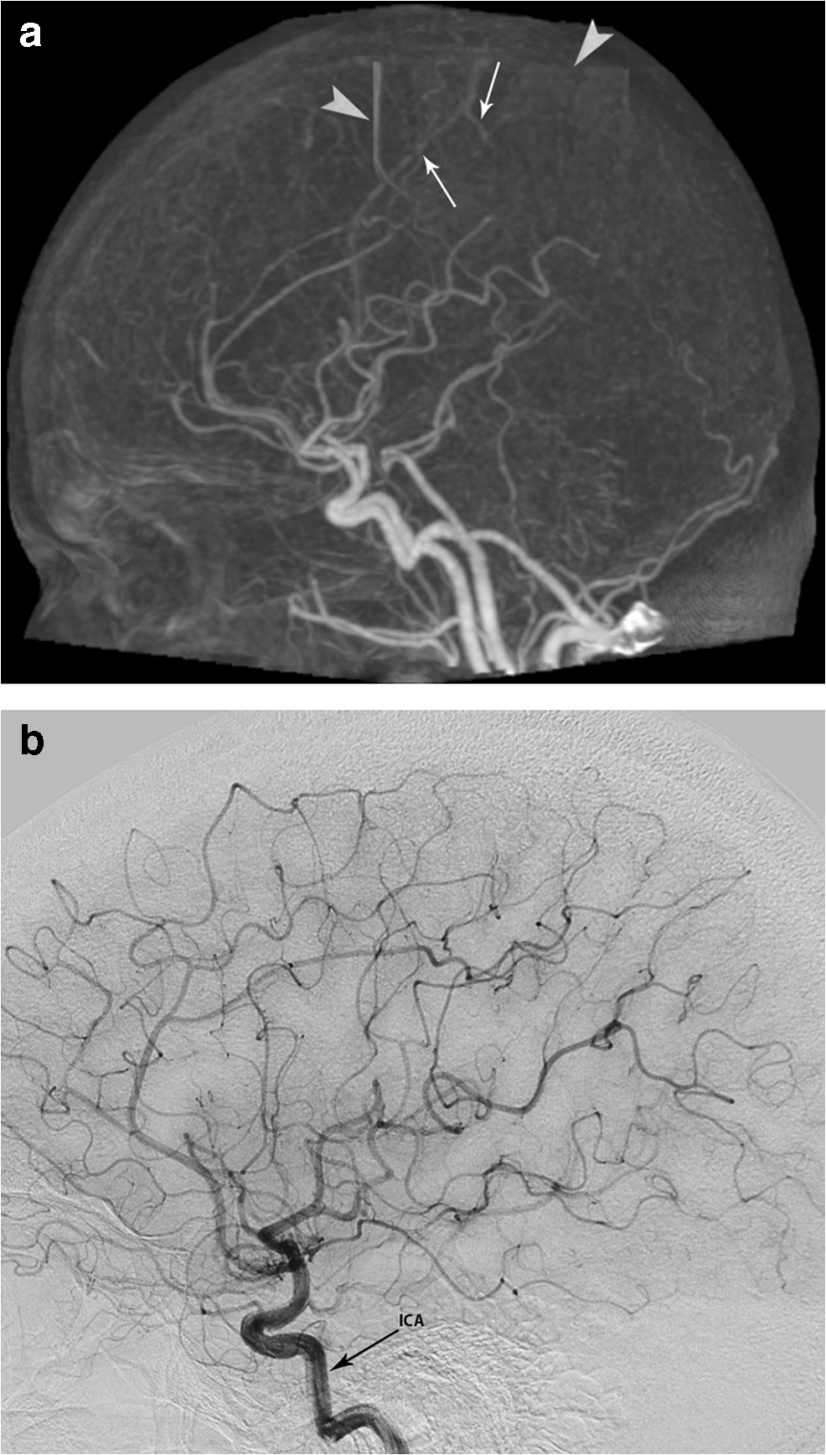
Panels **a** and **b** (right internal carotid artery injection, ICA) are from the case with a false-positive finding. 4D-CTA demonstrates motion artifacts (arrowheads). The arrows indicate veins that seem to appear early, suggesting the presence of an AV shunt, which is not corroborated by the DSA

Case 1 (false positive, Fig. 2): the 4D-CTA of a 52-year-old female, presenting with tinnitus, suggested a Borden-type I dAVF. 4D-CTA image quality was good with the diagnosis being considered sufficiently certain. It showed early venous filling in the late arterial phase in the right hemisphere which was not reproduced by DSA.

**Fig. 3:**
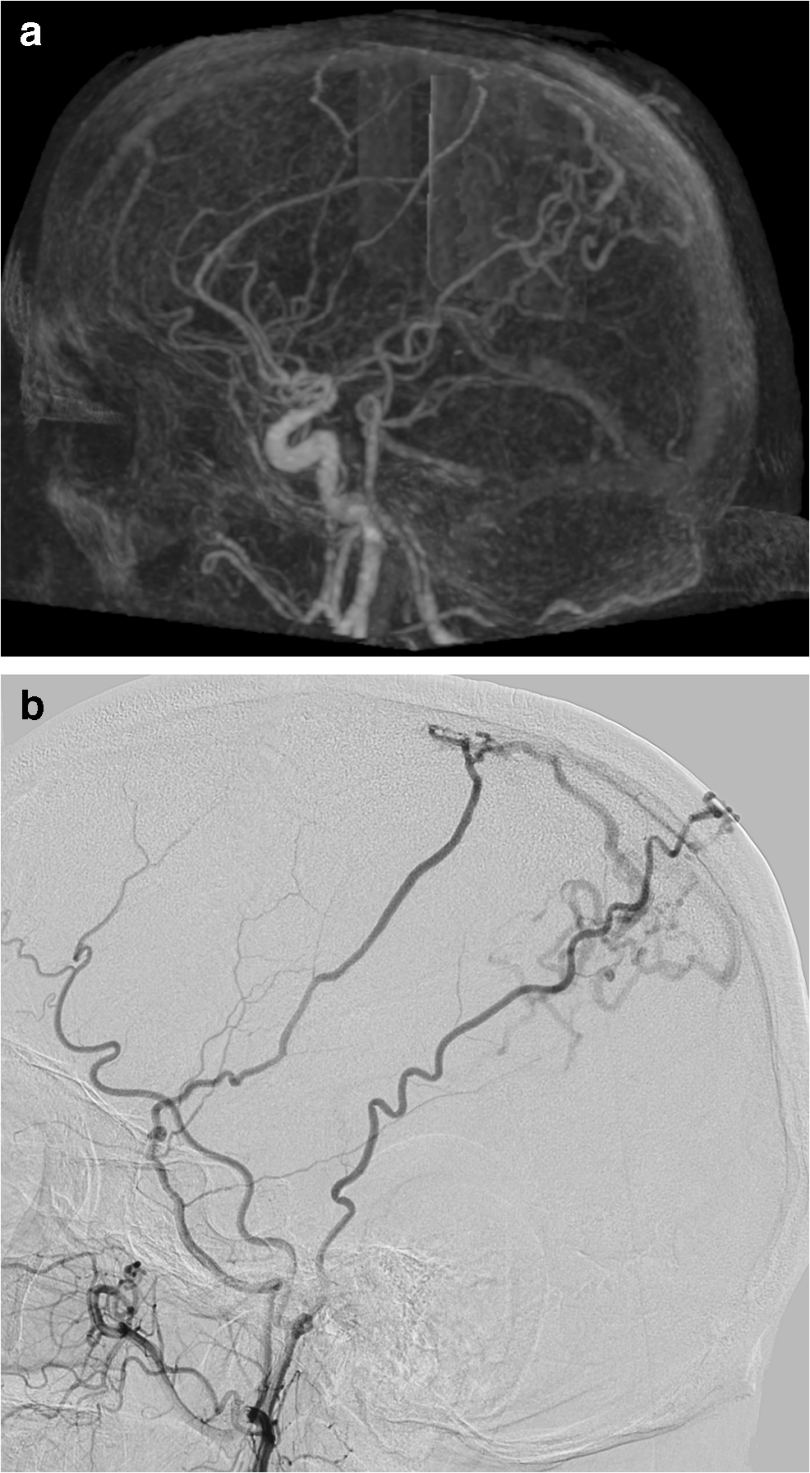
Panels **a** and **b** (right external carotid injection) are from the case with 4D-CTA misclassification. The early draining veins are clearly visible in both studies. They were thought to represent drainage from a bAVM with the 4D-CTA while DSA clearly demonstrates a dAVF

Case 2 (misclassification, Fig. 3): the 4D-CTA of a 59-year-old male, presenting with headache, was of poor image quality and showed early venous filling and appeared to demonstrate a bAVM, as an abnormal vascular pattern suggested the presence of a nidus. The 4D-CTA diagnosis was considered to be sufficiently certain. However, DSA demonstrated a Borden-type I dAVF.

**Fig. 4:**
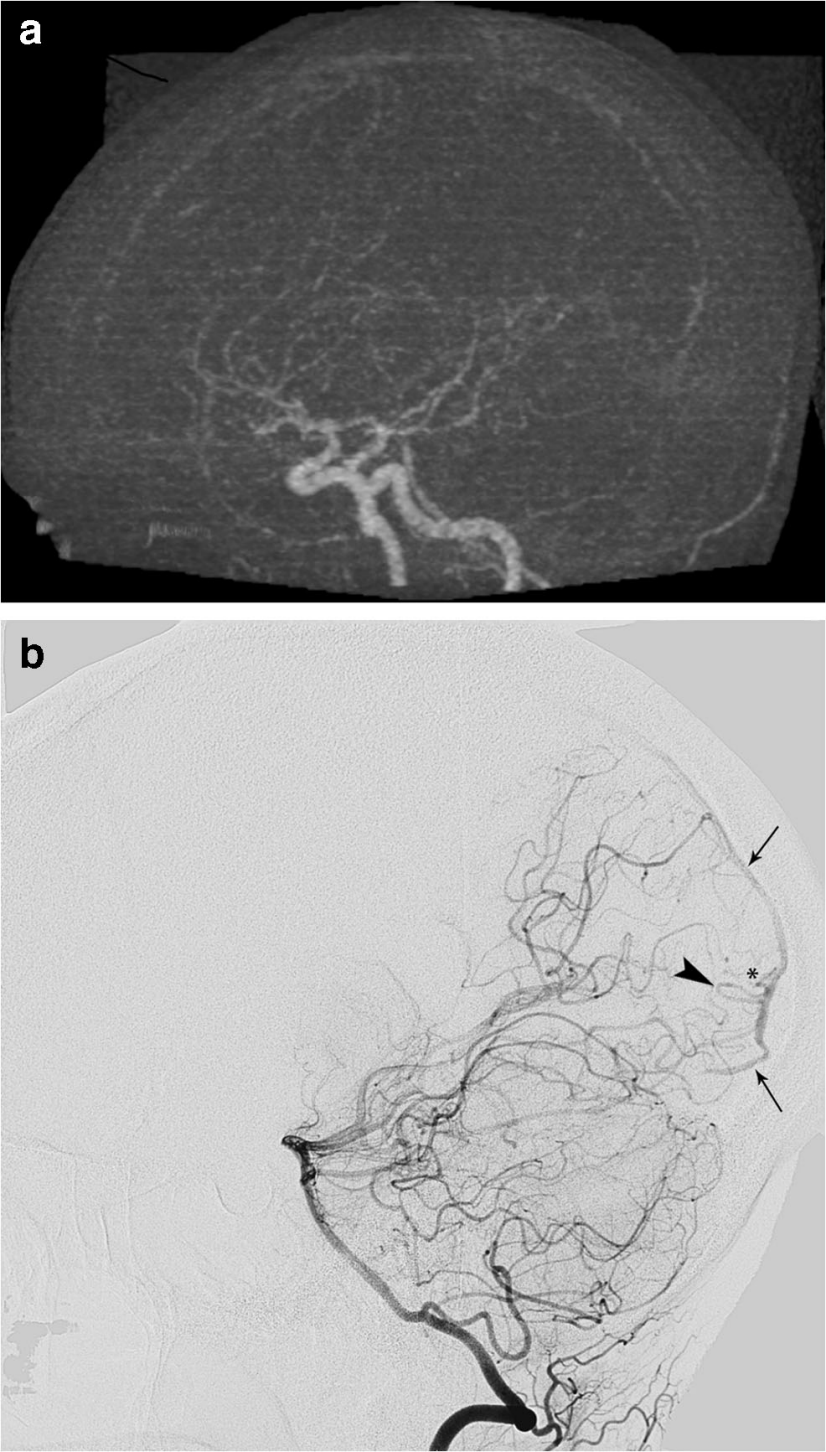
Panels **a** and **b** (left vertebral artery injection) are from the case where a false-negative 4D-CTA missed a micro-AVM. The DSA shows early venous filling (arrows). The arrowhead and asterisk respectively depict a feeding artery and the area of the shunt

Case 3 (false negative, Fig. 4): the 4D-CTA of a 29-year-old female, presenting with hemorrhage, was of good image quality and did not demonstrate an abnormality, although significant doubt was noted. DSA demonstrated a micro-AVM (< 3 cm) with multiple draining veins.

**Fig. 5:**
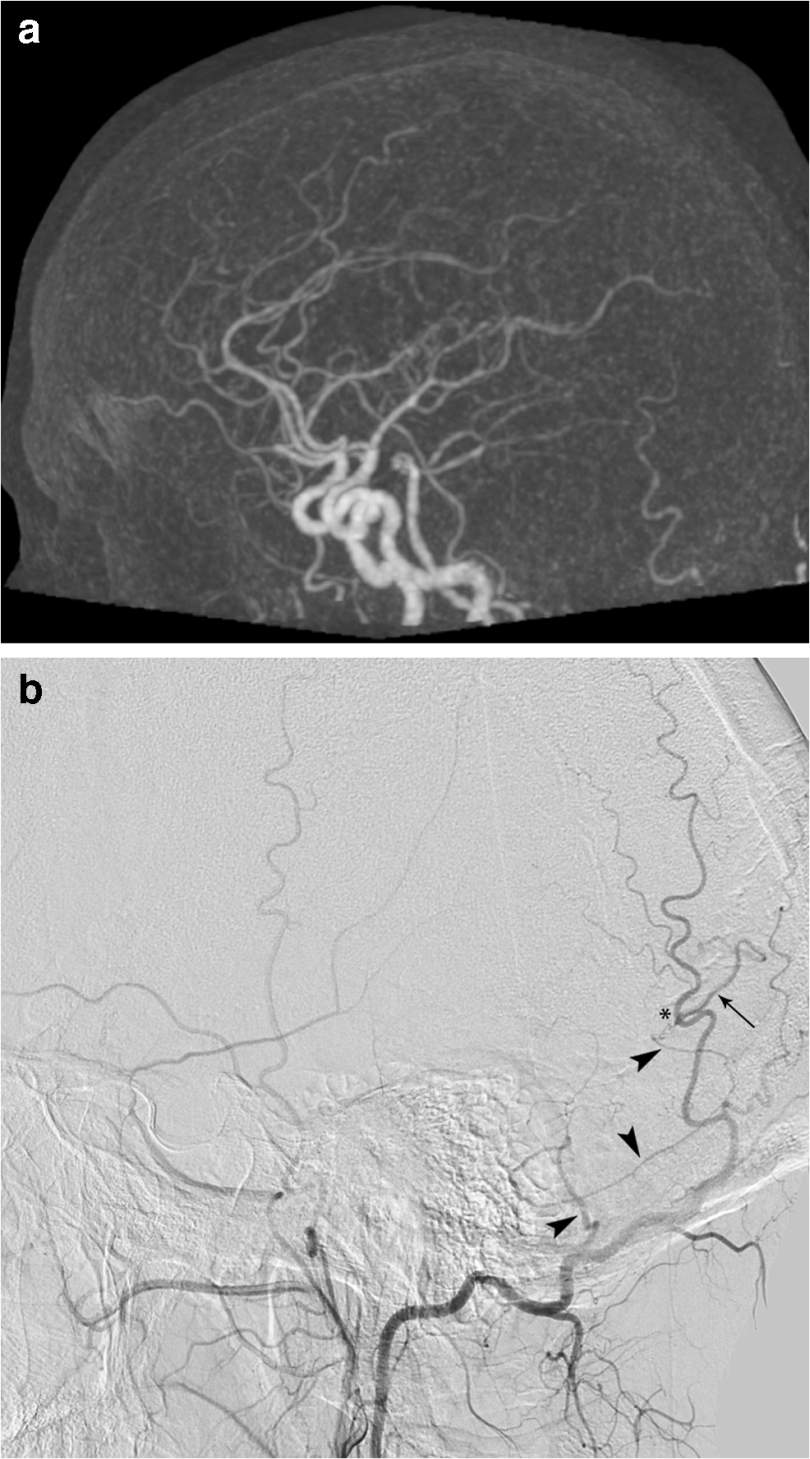
Panels **a** and **b** (right external carotid artery injection) are from the case where a false-negative 4D-CTA missed a dAVF. The DSA demonstrates a small transosseous dural branch from the occipital artery (arrowheads) feeding a Borden-type 3 dAVF (asterisk). The arrow depicts the arterialized draining vein. Some of these structures are partly seen in the 4D-CTA study, but their significance is unclear

Case 4 (false negative, Fig. 5): the 4D-CTA of a 31-year-old male, presenting with a seizure, was of moderate image quality and could not demonstrate an AV shunt. Significant doubt was noted and DSA subsequently demonstrated a Borden-type III dAVF.

### Interobserver analysis

Thirty cases were selected to represent the entire study population: 21 negative cases, 6 dAVFs, and 3 bAVMs. The 4D-CTAs from these cases were independently read a second time. Regarding diagnosis (no diagnosis, dAVF, and bAVM), the observers agreed in 29 cases (97%, kappa = 0.925). The one case reported differently had a Borden-type III dAVF that was correctly reported by one reader and missed by the other. Both readers reported the image quality to be poor and both reported significant doubt regarding the diagnosis with 4D-CTA. This was due to movement artifacts and poor timing of the contrast bolus.

Regarding Borden and Spetzler-Martin classifications, both observers agreed in all cases (kappa = 1.00).

## Discussion

In this prospective cohort study, 4D-CTA proved to have high diagnostic accuracy for detecting cranial arteriovenous shunts, supporting its use as a non-invasive alternative to DSA. We demonstrated that 4D-CTA has high sensitivity (93%) and specificity (98%) for detecting shunting lesions and that all false-negative results were characterized by significant doubt regarding the diagnosis.

Since all arteriovenous shunts lead to early venous filling, it is this feature that is looked for in the angiographic images to determine whether a shunt exists. Once the early venous filling is detected, features such as appearance of a nidus, the location of the shunt, and the apparent feeders will help to determine whether the lesion matches a dAVF or a bAVM. Although 4D-CTA misdiagnosed over 10% of the AV shunts (3 out of 28; 10.7%), in one of these, a shunt was found on 4D-CTA but erroneously diagnosed as a bAVM instead of a dAVF. The other two were truly false negative but read with significant doubt. Significant doubt was recorded in 6.6% of cases and should prompt additional imaging. In other words, if 4D-CTA is used as a diagnostic tool when an AV shunt is suspected, there is a small chance (6.6%) that doubt regarding the diagnosis persists and DSA is still warranted. In the remaining majority, all lesions will be found, but with a very small chance (1.4%) that 4D-CTA will not interpret the type of shunt correctly.

Although in the lesions detected with 4D-CTA, 4D-CTA agreed with DSA regarding Borden or Spetzler-Martin classification, DSA is more accurate in the evaluation of angioarchitectural details. This is due to selective vessel injection and a higher spatial and temporal resolution with DSA. Although 4D-CTA resolution may improve with future developments, non-selective vessel opacification is a fundamental characteristic of this modality and will, thus, always hamper image interpretation to some extent. On the other hand, 4D-CTA adds time-resolved cross-sectional imaging and also visualizes non-vascular tissues, which may prove beneficial during surgery, especially as it may be used in frameless stereotaxy. In earlier studies, we demonstrated these differences between 4D-CTA and DSA in patients known to have a dAVF or bAVM [15–18]. Other authors also reported on the use of 4D-CTA in both spinal and cranial arteriovenous shunts [21, 22]. However, in all of these studies, only patients who were already diagnosed with such a lesion were examined. The present study not only corroborates those findings, but also demonstrates the diagnostic capabilities of 4D-CTA in a patient population suspected, rather than known, to have a bAVM or dAVF. Thus, this is the first demonstration of the true value of 4D-CTA as a diagnostic tool.

Based on these results, in centers with similar imaging technology available, an additional DSA could be reserved for cases when there is significant doubt regarding the 4D-CTA diagnosis (6.6% of all cases) or when a dAVF or bAVM has been found by 4D-CTA, to further determine its precise angioarchitectural details and allow treatment planning. In our population, this would have resulted in the avoidance of a DSA in almost two-thirds (62%) of cases. An even higher number of cases would not require a DSA if it is omitted when treatment is not being considered, e.g., based on the lesion’s classification or other clinical details. Finally, endovascular treatment will allow DSA visualization at the beginning of the procedure, again sparing the patient an additional diagnostic DSA session. This reduction in DSA studies is important as 4D-CTA is less invasive, less expensive, and less time-consuming and does not carry the risk of neurological complications.

Despite the high sensitivity and specificity, two shunting lesions were not detected by 4D-CTA: one dAVF and one bAVM (false-negative 4D-CTA results). Not having discovered such a lesion might have had great consequences for the patients involved, as both lesions represented a risk of intracranial hemorrhage. Index of suspicion was high in both cases, as the dAVF presented with hemorrhage and the bAVM with a neurological symptom (seizure). Using the above reasoning, both would have required an additional DSA due to significant doubt regarding the 4D-CTA diagnosis. However, one could also argue that a case with such a high index of suspicion deserves a DSA, rather than or supplementary to a 4D-CTA, anyway.

Furthermore, a high interobserver agreement was found regarding the presence of a dAVF or bAVM and its classification. The single case with disagreement between the two readers again underlines the need for additional imaging when image quality is poor and significant doubt regarding the diagnosis persists.

With regard to radiation dose, it is not easy to compare the dose of 4D-CTA with that of DSA, since the dose received during these studies depends on many variables. In an earlier publication, we reported the radiation dose of diagnostic cerebral DSA to lie between 7.9 and 9.1 mSv [16]. As presented in our methods, different 4D-CTA acquisition protocols were used in our study with the tradition dose varying between 5.2 and 11.5 mSv. This depended mostly on the rotation speed during the continuous acquisition phase.

There are several limitations to the present study. Although we intended to include an unselected population suspected of having a dAVF or bAVM, this did not appear entirely feasible. The protocol dictated that the 4D-CTA and DSA were not performed on the same day due to the cumulative contrast burden. Thus, in emergency cases, inclusion was sometimes thought to introduce an unacceptable delay in diagnosis and treatment. This may have resulted in a skewed distribution of presenting symptoms, with less intracranial hemorrhages and more non-emergency presentations such as pulsatile tinnitus. Furthermore, the protocol did not require any non-dynamic imaging, such as single-phase CTA or MRI/MRA, to be acquired prior to inclusion. Therefore, we cannot draw conclusions regarding the value of 4D-CTA versus such modalities. Moreover, the protocol excluded patients with prior time-resolved imaging, since we were interested in newly suspected cases. Thus, any comparison between 4D-CTA and such time-resolved modalities, e.g., trMRA, also lies beyond the scope of this study. Finally, it would be of interest to use 4D-CTA in other patient categories as well, e.g., as repeat imaging modality in patients with a wait-and-scan policy or to check for therapy efficacy after surgery or endovascular treatment. Although such use of 4D-CTA appears attractive, our results cannot be extrapolated to these patient categories.

## Conclusion

4D-CTA is a less invasive dynamic imaging tool than DSA, with high sensitivity, specificity, and interobserver reliability in the detection and classification of intracranial arteriovenous shunts. Although, in rare occasions, false negatives may occur, in our population, these cases were all characterized by significant doubt regarding the 4D-CTA diagnosis. Taking this into account, 4D-CTA could have replaced DSA in the majority of our study population. Our study provides evidence that 4D-CTA is an alternative to DSA as a first modality in the diagnostic workup of patients suspected of having a dAVF or bAVM.

## Abbreviations

4D-CTA: Time-resolved CTA
bAVM: Brain arteriovenous malformation
dAVF: Dural arteriovenous fistula
DSA: Digital subtraction angiography
ECA: External carotid artery
ICA: Internal carotid artery
MRA: MR angiography
SM: Spetzler-Martin
VA: Vertebral artery
